# Design and Development of Sc_0.2_Al_0.8_N-Based Dual-Piezoelectric-Layer MEMS Hydrophone

**DOI:** 10.3390/mi17020235

**Published:** 2026-02-11

**Authors:** Danfeng Cui, Xiaoya Duan, Ziyue Guan, Ningyuan Hu, Yikun Guo, Yiming Yao, Haojie Yan, Chenyang Xue

**Affiliations:** 1 School of Intelligent Manufacturing and Control Engineering, Shanghai Polytechnic University, Shanghai 201209, China; 20251513167@sspu.edu.cn (Y.Y.); 20251513227@sspu.edu.cn (H.Y.); 2 State Key Laboratory of Dynamic Measurement Technology, School of Instrument and Electronics, North University of China, Taiyuan 030051, China; xiaoyad1005@gmail.com (X.D.); guanzy0115@163.com (Z.G.); huningyuan@nuc.edu.cn (N.H.); guoyikun@nuc.edu.cn (Y.G.); xuechenyang@nuc.edu.cn (C.X.)

**Keywords:** hydrophone, AlN/ScAlN, piezoelectric, equivalent noise density

## Abstract

An innovative design for a dual-piezoelectric-layer MEMS hydrophone based on a composite film of scandium-doped aluminum nitride (Sc_0.2_Al_0.8_N) is presented. By designing the dual piezoelectric layer, the frequency response range has been expanded and the sensitivity of the device has been significantly enhanced. Meanwhile, doping with scandium can significantly increase the piezoelectric coefficient, enhancing the sensitivity. According to the standard underwater acoustic calibration test, the device exhibits an average sound pressure sensitivity of −162 dB (re: 1 V/μPa) across the 20 Hz–50 KHz frequency band and equivalent noise density of 47 dB (re: 1 μPa/√Hz) with a linearity of 99%. The experimental results show that the comprehensive performance of the dual-piezoelectric-layer hydrophone provides a new solution for underwater sensing and detection, and opens up a new path for the performance optimization of passive sonar systems.

## 1. Introduction

As critical components in underwater acoustic detection, hydrophones have become increasingly integral to applications in underwater acoustic communication, naval operations, underwater imaging, positioning, and sonar systems [1,2,3,4,5,6,7]. With the evolution of hydrophones across various applications, there is a growing demand for enhanced sensitivity, reduced equivalent noise density (END), superior linearity, and further miniaturization.

Large hydrophones based on piezoelectric ceramics currently lead the market [8,9,10]. Yet, their considerable size, intricate manufacturing processes, inconsistent uniformity, and high costs limit their practical applications [10,11,12]. The advancement of Micro-Electro-Mechanical Systems (MEMSs) technology has catalyzed a shift toward miniaturization and integration in hydrophone design [12,13,14].

The commonly used piezoelectric film materials in MEMS piezoelectric hydrophones include lead zirconate titanate (PZT), zinc oxide (ZnO), and aluminum nitride (AlN). PZT has a high piezoelectric coefficient, but it has relatively high power consumption and is incompatible with CMOS processes. ZnO films are susceptible to temperature changes and have relatively weak corrosion resistance, making them difficult to operate in harsh environments. Aluminum nitride (AlN) has gained prominence as an optimal material for piezoelectric MEMS hydrophones due to its full compatibility with CMOS fabrication and suitability for mass production [15,16,17]. Additionally, the quality factor (i.e., the ratio of the piezoelectric coefficient to the dielectric constant) of AlN is superior to that of PZT. Meanwhile, the thickness of the AlN film is uniform and has a low acoustic impedance, which helps to reduce the internal dielectric loss of the device and thereby improve the signal-to-noise ratio, meeting the demand of MEMS hydrophones for highly sensitive detection of very low-frequency signals.

Scandium aluminum nitride (ScAlN), known for its softer structure and higher electronegativity, achieves a higher piezoelectric constant than aluminum nitride (AlN) [18]. Table 1 lists key material properties, such as the thin-film piezoelectric coefficient e_31,f_ and the relative dielectric constant ε_33_, for commonly utilized piezoelectric layers, including lead zirconate titanate (PZT), AlN, 9.5% doped ScAlN, and 20% doped ScAlN. The sound pressure of piezoelectric MEMS hydrophone is proportional to e_31,f_/ε_33_ [19], indicating that the MEMS hydrophone with 20% ScAlN has superior piezoelectric constants and acoustic pressure sensitivity. Furthermore, the dual-electrode design, incorporating a differential configuration readout circuit, achieves a sound pressure sensitivity higher than a single-ended configuration under similar conditions [20].

This paper presents the design and experimental validation of a dual-piezoelectric- layer MEMS hydrophone based on Sc_0.2_Al_0.8_N. In view of the low piezoelectric coefficient and limited sensitivity of AlN-based MEMS hydrophones, this paper proposes to increase the piezoelectric coefficient by scandium doping (20%), and adopts a dual piezoelectric layer structure and differential reading method to enhance the sensitivity and detection capability of the hydrophones in low-frequency passive sonar systems. In water, the hydrophone attains a sensitivity of −162 dB (re: 1 V/μPa) in the range of 20–50 kHz, and an equivalent noise density of 55 dB (re: 1 μPa/√Hz) at 1 kHz with a linearity of 99%.

## 2. Design

**Figure 1:**
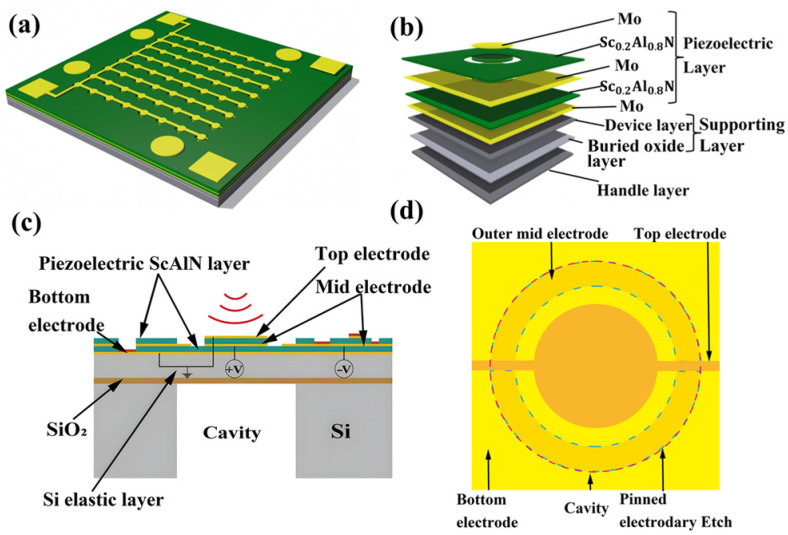
(**a**) Schematic diagram of the hydrophone; (**b**) single cell of the 8 × 8 array; (**c**) cross-sectional view of a single cell; (**d**) top view of a single cell.

The system studied the effects of geometric structure, AlN thickness and electrode shape on the performance of the hydrophone. Figure 1a shows the schematic diagram of an 8 × 8 array of hydrophones. As shown in Figure 1b, a single cell is composed of a piezoelectric layer (Mo-Sc_0.2_Al_0.8_N-Mo-Sc_0.2_Al_0.8_N-Mo), supporting layer and handle layer. The cross-sectional and top views are depicted in Figure 1c and Figure 1d, respectively.

The resonant frequency and sensitivity of a hydrophone depends on the material properties and geometric parameters, including the Young’s modulus, density, radius and thickness of the film [12]. The resonant frequency $f_{0}$ of a sensing cell in air can be expressed as [16]

(1) $$f_{0}=\frac{\lambda_{ij}^{2}}{2\pi \times d^{2}}\sqrt{\frac{E_{eq}}{12\times \rho_{eq}\times (1-\upsilon_{eq}^{2})}}$$

where d is the diameter of the circle inscribed in the hexagonal film. $E_{eq}$, $\rho_{eq}$, and $\upsilon_{eq}$ are the equivalent elastic modulus, equivalent density, and equivalent Poisson’s ratio, respectively. The eigenvalues of the first nine modes $\lambda_{ij}$ for a circular film are listed in Table 2 [12].

Due to the influence of liquid damping, the resonance frequency of the hydrophone in a liquid environment is lower than that in the air, which can be approximately expressed as [12,13]:

(2) $$f_{0,fluid}=f_{0}{\left(1+0.67a\rho_{fluid}/\rho \right)}^{-0.5}$$

where $\rho_{fluid}$ and ρ denote the fluid density and air density, respectively. The sound pressure sensitivity S of a piezoelectric hydrophone can be calculated as:

(3) $$S=\frac{d_{31}t_{p}}{A_{e}\varepsilon p}\iint_{A_{e}}(\sigma_{r}+\sigma_{\theta })\mathrm{dS}$$

where $A_{e}$ denotes the area of the top electrode; ε and $d_{31}$ are the permittivity and piezoelectric strain coefficient of the piezoelectric film, respectively.

**Figure 2:**
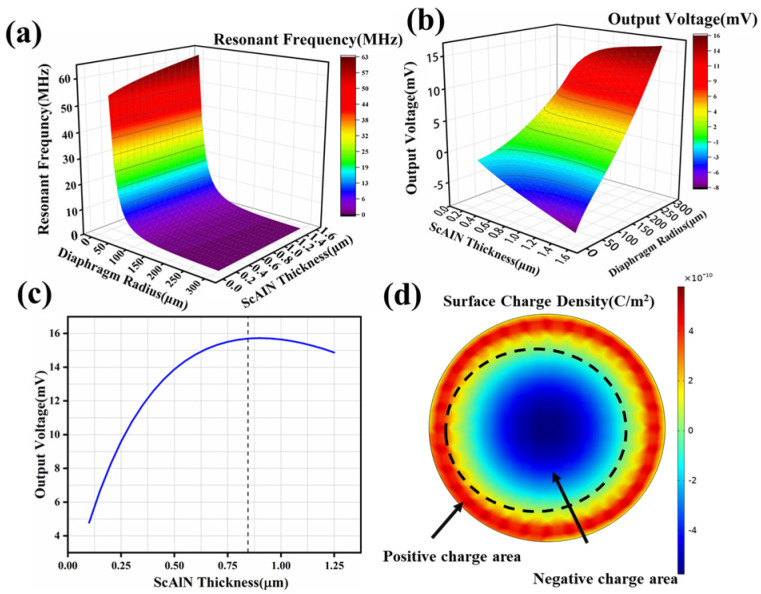
(**a**) Resonant frequency versus film radius and ScAlN layer thickness; (**b**) output voltages versus film radius and ScAlN layer thickness; (**c**) relationship between the thickness of ScAlN and the output voltage; (**d**) hexagonal membrane surface charge density distribution.

The finite element analysis (FEA) is used to investigate the relationship between the resonant frequency and the thickness and radius values of ScAlN, as shown in Figure 2a. The output voltages obtained at different radius values and thickness are shown in Figure 2b. It is observed that the resonant frequency is directly proportional to the thickness and inversely proportional to the radius, with the radius having a greater effect on the resonant frequency. The output voltage is directly proportional to both the thickness and radius.

The finite element analysis (FEA) is used to investigate the relationship between the resonant frequency and the thickness and radius valuesgeometric parameters of ScAlN, is investigated using the finite element analysis (FEA), as shown in Figure 2a. The output voltages obtained at different radius values and thickness are shown in Figure 2b. It is observed that the resonant frequency is inversely proportional to the radius and directly proportional to the thickness and inversely proportional to the ra-dius, with the radius having a greater effect on the resonant frequency. The output voltage is directly proportional to both the thickness and radius.

The larger radius hydrophones can offer lower resonant frequencies, making them more suitable for underwater low-frequency detection, the resonant frequency should be as low as possible. This requires a larger radius. However, considering the imple-mentation of MEMS technology, a radius of $r_{1}=150$ μm was chosen and the impact of ScAlN thickness on the output voltage is depicted in Figure 2c, where the maximum output voltage occurs at a thickness of 0.8 μm

Furthermore, there is a correlation between the top electrode radius $r_{2}$ and the film radius $r_{1}$. When the incident pressure acts on the ScAlN layer, due to the positive piezoelectric effect, charges will be generated on the ScAlN surface. The internal (near the center) and external charges have opposite polarities, as shown in Figure 2d. Therefore, the top electrode should cover an area without stress and neutral charges to avoid canceling out the opposite-polarity charges and maximizing the output voltage at approximately 70% of the radius $r_{1}$, $\sigma_{r}+\sigma_{\theta }=0$, where $\sigma_{r}$ and $\sigma_{\theta }$ represent the ra-dial and tangential stresses respectively. Therefore, $r_{2}=70\%r_{1}$ is the optimal electrode radius [23].

## 3. Fabrication and Package

**Figure 3:**
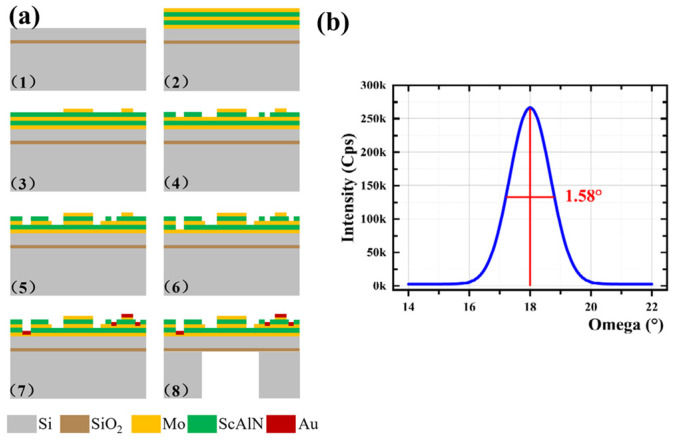
(**a**) Fabrication process of the ScAlN-based hydrophone; (**b**) X-ray diffraction (XRD) spectra of the ScAlN film.

Figure 3a illustrates the fabrication process of the ScAlN-based MEMS hydrophone.

(1) The MEMS manufacturing initiates with a double-side-polished, insulator-on-silicon (SOI) wafer. (2) For the drive layer formation, magnetron sputtering sequentially deposits a bottom electrode of Mo, ScAlN, a middle electrode of Mo, additional ScAlN, and finally a top electrode of Mo. (3) The top Mo film then undergoes ion beam etching (IBE) to establish the top electrode. (4) The initial piezoelectric layer of ScAlN is dry-etched with chlorine gas to generate a quasi-pinned pattern. (5) IBE of the middle Mo electrode follows, forming internal and external middle electrodes for charge collection. (6) The second ScAlN piezoelectric layer is similarly dry-etched with chlorine gas to free the bottom electrode. (7) The process continues with the evaporation of 50 nm Ti and 100 nm Au, succeeded by a lift-off process to create contact pads. (8) Last, the vibrating membrane is released through deep reactive ion etching (DRIE), using the buried oxide (BOX) as the etch stop layer.

The high-quality preferred orientation of ScAlN can enhance the piezoelectric response and the sensitivity of the device. The X-ray diffraction (XRD) rocking curve of the ScAlN films deposited for this project, as illustrated in Figure 3b, reveals a maximum full width at half maximum (FWHM) of approximately 1.58°. It indicates that the ScAlN film is highly oriented along the C-axis and has excellent crystalline quality.

**Figure 4:**
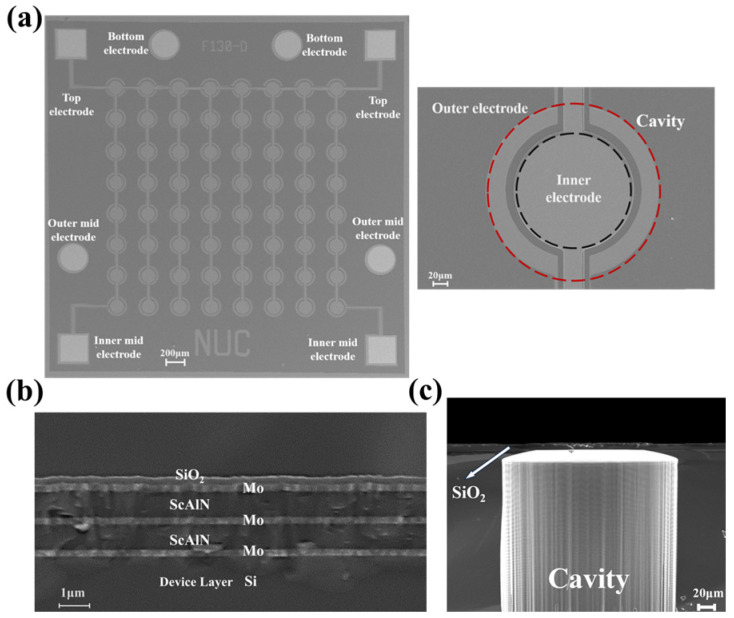
(**a**) Scanning electron microscope (SEM) image showing the hydrophone array and the unit; (**b**) SEM image of the material stacking layer; (**c**) cross-sectional view of the cavity after DRIE.

Figure 4a shows the array and unit scanning electron microscope (SEM) images captured using the TESCAN MIRA3. The sensing film is predominantly composed of a Mo/Sc_0.2_Al_0.8_N/Mo/Sc_0.2_Al_0.8_N/Mo with thickness values of 0.2 μm/0.8 μm/0.2 μm/0.8 μm as shown as Figure 4b. Additionally, Figure 4c is the cross-sectional view of the cavity following deep reactive ion etching (DRIE), featuring a back cavity thickness of 300 μm etched to the SiO_2_ stop layer. Notably, a residual 6.13 μm of Si at the bottom edge remains unetched, as further etching could potentially damage the vibrating membrane.

**Figure 5:**
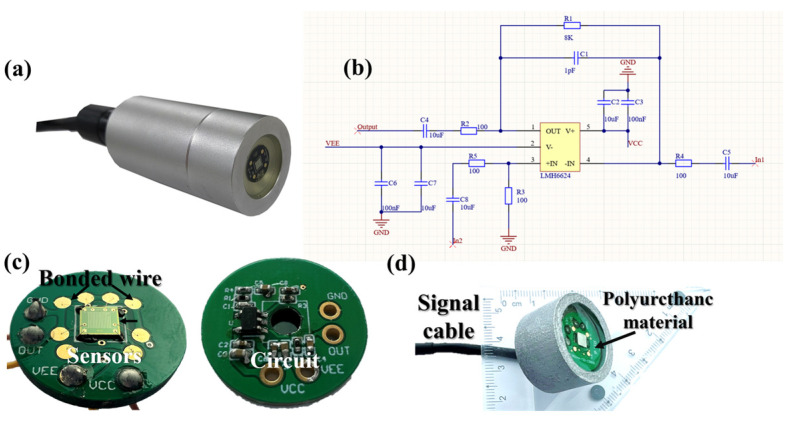
Encapsulation structure of the MEMS hydrophone. (**a**) Encapsulated hydrophone; (**b**) electric schematics; (**c**) PCB with a MEMS hydrophone; (**d**) packaged hydrophone.

The encapsulation structure of the hydrophone is shown in Figure 5a. When sound travels through different media, it will experience attenuation, which is a key consideration in packaging design. The fabricated sensor chip, along with its readout circuit (Figure 5b), is die-attached to a printed circuit board (PCB) (Figure 5c). Figure 5d illustrates the hydrophone, encapsulated with acoustic transparent polyurethane material. Sound wave reflections at the interface between the hydrophone’s matching layer and water occur due to impedance mismatch. Due to the excellent sound permeability, low water permeability, and seawater immersion resistance of polyurethane (PU), it is selected as the impedance-matching material to reduce this impedance disparity [24], achieving a reflectivity of 2.74%.

The encapsulation process is as follows:

(1) Fix the PCB in the groove of the cylindrical aluminum alloy and secure it with UV glue. (2) The signal cables are connected through the aviation connector at the tail end face, ensuring the stability of signal transmission. (3) A certain thickness of JA-2S polyurethane (Shanxi Keying Technology, Taiyuan, China) material is injected at the front end, and the vacuum encapsulation process is used to eliminate air bubbles, ensuring the uniform distribution of the material and its close adhesion to the surface of the device. (4) The material is continuously vulcanized for 24 h until it naturally cools, forming a high-strength acoustic matching layer.

## 4. Results

### 4.1. Electrical Characterization

The effective electromechanical coupling coefficient $K_{eff}^{2}$ is a key physical parameter quantifying the efficiency of energy conversion between electrical and mechanical energy in a sensor [25,26]. Its calculation is based on Equation (4) [27]:

(4) $$K_{eff}^{2}=1-{\left(\frac{f_{r}}{f_{a}}\right)}^{2}$$

where $f_{r}$ and $f_{a}$ are the resonant and anti-resonant frequencies, respectively.

**Figure 6:**
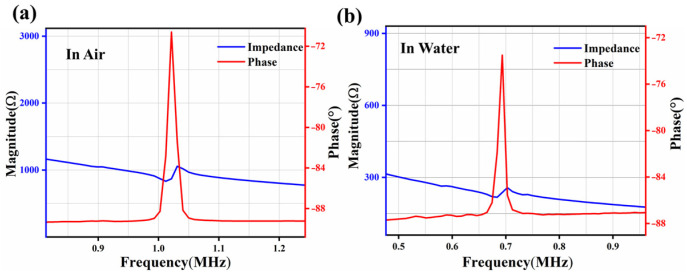
The impedance curves of the hydrophone (**a**) in air and (**b**) DI water.

The impedance spectrum, which includes $f_{r}$ and $f_{a}$ values, is measured using the KEYSIGHT E4990A (Keysight Technologies, Santa Rosa, CA, USA) impedance analyzer. As depicted in Figure 6a, the first-order resonant frequency is about 1.015 MHz, and the anti-resonant frequency is about 1.03 MHz, with the electromechanical coupling coefficient calculated to be 3% according to (4). The performance of the hydrophone in deionized (DI) water is shown in Figure 6b. Due to the mass effect [28], the resonant frequency is reduced to 0.68 MHz and the anti-resonant frequency is approximately 0.7 MHz. The resulting effective electromechanical coupling coefficient is determined to be 5.64% in DI water.

### 4.2. Acoustic Characterization

**Figure 7:**
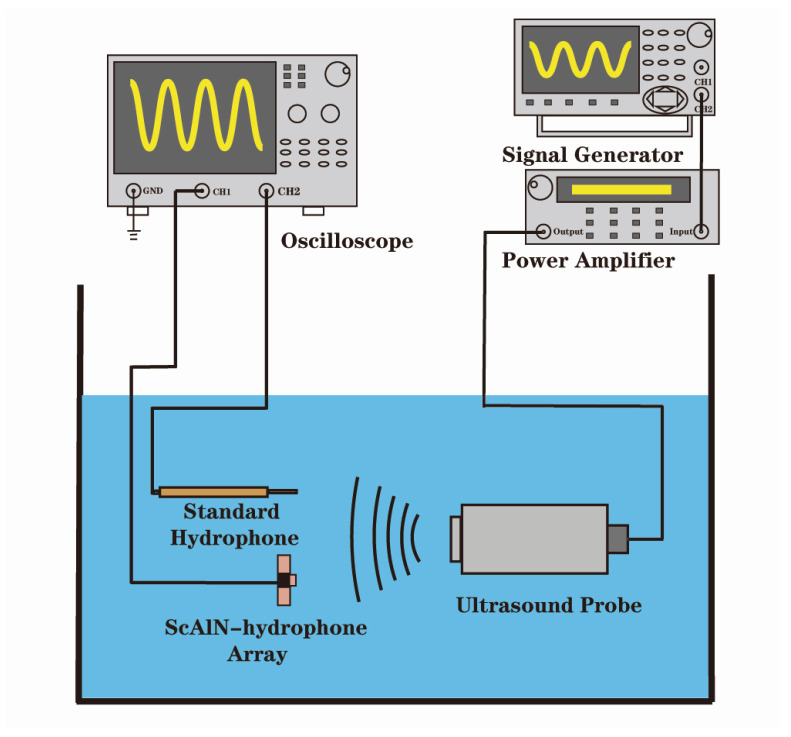
Underwater test platform.

Figure 7 shows an underwater test platform. The emission transducer is motivated by the signal generator. Under constant sound pressure excitation, the sensitivity of the standard hydrophone $S_{ref}$ is proportional to that of the test hydrophone $S_{test}$, and this proportion is equal to the ratio of the output voltage of the standard hydrophone $U_{ref}\;$ to that of the test hydrophone $U_{test}$ [29]:

(5) $$S_{test}=\frac{U_{test}}{U_{ref}}\cdot S_{ref}$$

Compared with other calibration methods, the comparative calibration method has the advantages of simple operation and high efficiency. The specific test steps are as follows:

(1) The signal generator generates a signal with frequency *f* to excite the sound source; (2) the electrical signal converted by the hydrophone is amplified and filtered before being displayed; and (3) test points are set successively within the frequency band, and the sound pressure is controlled by changing the amplitude or frequency of the signal generated by the signal generator. Repeat steps 1 and 2 to test the sensitivity and linearity of the hydrophone.

**Figure 8:**
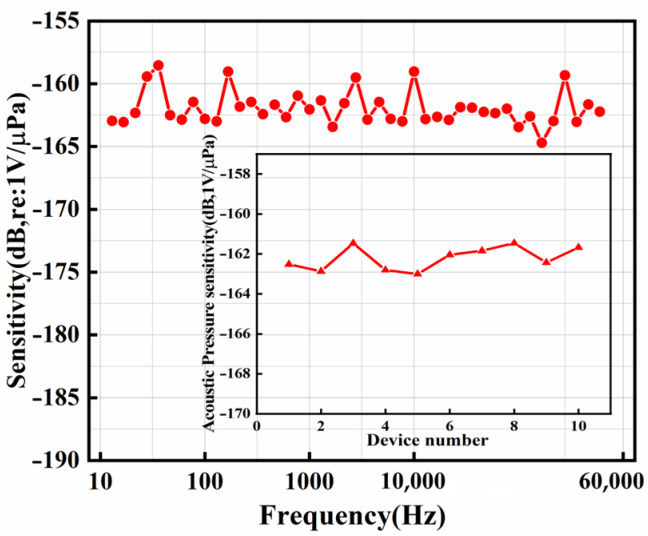
Sensitivity curve of the hydrophone under different frequencies. Sensitivity testing of multiple devices (inset).

Figure 8 showcases the measured sensitivity curve of the ScAlN-based MEMS hydrophone across various frequencies in water. The average sensitivity is −162 dB (re: 1 V/μPa), demonstrating a flat response from 20 Hz to 50 kHz. To ensure data integrity, measurements from ten devices fabricated in the same batch are reported and the average variance in acoustic sensitivity among different hydrophones of the same structure is around 2 dB. This clearly indicates that the hydrophones fabricated in this batch have excellent consistency.

**Figure 9:**
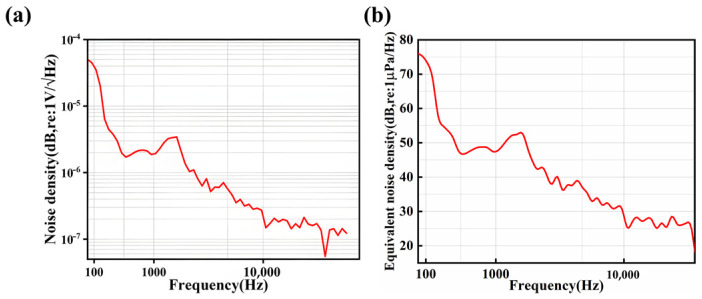
The measured (**a**) noise resolution and (**b**) equivalent noise resolution of the hydrophone.

The main overall noise sources of the test include dielectric loss, input noise voltage and current of the preamplifier, as well asJohnson (thermal) noise [30]. The test is conducted using an Agilent 35670A dynamic signal analyzer. The measured noise resolution is shown in Figure 9a. There is a relationship between sound pressure sensitivity, noise density and equivalent noise resolution [20,30]:

(6) $$Equivalent\;noise\;resolution=\frac{Noise\;density}{Acoustic\;pressure\;sensitivity}$$

The corresponding equivalent noise resolution is calculated using Equation (6) as shown in Figure 9b, and the equivalent noise density (END) of the MEMS hydrophone at 1 kHz is 55 dB (re: 1 μPa/√Hz).

**Figure 10:**
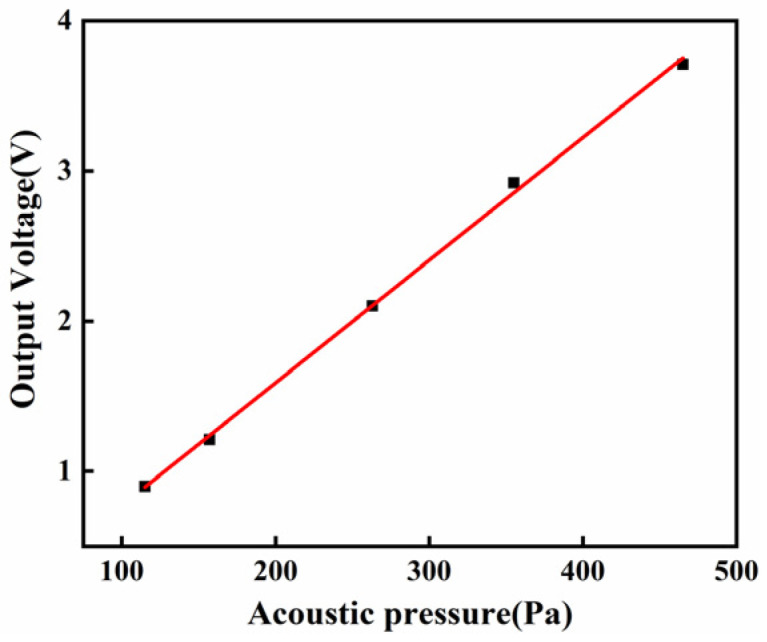
The linearity values in water at 1000 Hz.

Another crucial indicator of a hydrophone’s performance is linearity, which delineates the relationship between input sound pressure and the output voltage of the hydrophone. The linearity value is about 99%, as shown in Figure 10. These results lay a solid groundwork for the practical application of hydrophones.

Table 3 presents a performance comparison between the reported MEMS hydrophones and commercial hydrophones. When contrasted with advanced conventional bulk piezoceramic hydrophones and other piezoelectric MEMS hydrophones in the literature, ScAlN-based MEMS hydrophones demonstrate a wider frequency response, higher sensitivity and optimal equivalent noise density.

## 5. Conclusions

This paper reported on a novel MEMS hydrophone based on Sc_0.2_Al_0.8_N featuring a dual piezoelectric layer. The bimorph design is utilized to expand bandwidth, while approaches such as Sc doping, differential readout, array structure, and electrode size are employed to increase the charge in the receiving mode and enhance the output performance. The fabricated hydrophone achieved an average sensitivity of −162 dB (re: 1 V/μPa) in the range of 20 Hz to 50 kHz, and an equivalent noise density of 55 dB (re: 1 μPa/√Hz) at 1 kHz. The linearity value is about 99% in water. This research indicated that this device provides a promising approach for the development of next-generation hydrophones.

## Figures and Tables

**Table 1:** Material properties of commonly used piezoelectric layers.

Property	PZT [21]	AlN [21]	ScAlN (9.5% Doped) [21]	ScAlN (20% Doped) [22]
e_31,f_ (C/m^2^)	−10	−1.06	−1.79	−1.6
ε_33_	1200	9.5	10.5	6.5
e_31,f_/ε_33_ (C/m^2^)	−0.008	−0.112	−0.171	−0.246
d_31_ (pC/N)	−123	−2.181		−4.18
d_33_ (pC/N)	289	5.268		10.818

**Table 2:** Eigenvalues $\lambda_{ij}^{2}$ of the first nine modes.

$\lambda_{ij}^{2}$	*i =* 0	1	2
*j* = 0	10.32	21.26	34.88
1	39.77	60.82	84.58
2	89.1	120.1	153.8

**Table 3:** Performance comparison of the Sc_0.2_Al_0.8_N-based hydrophone with previously reported hydrophones.

Hydrophone	Cetacean CR2 [10]	Brüel & Kjær 8103 [11]	S. Shi et al. [31]	M. Sung et al. [32]	J. Xu et al. [14]	D. Yang et al. [5]	This Work
Technology	Piezoceramic	Piezoceramic	PZT	PZT	MEMS (AlN)	MEMS (AlN)	MEMS (Sc_0.2_Al_0.8_N)
Bandwidth (Hz)	1.9–28,000	3–20,000	20–2000	50–1000	10–8000	100–1600	10–50,000
Sensitivity (dB, re: 1 V/μPa)	−182.5 ± 0.3	−178	−189.3	−191.5 ± 1	−180 ± 1	−178	−162
END (dB, re: 1 μPa/√Hz)	68 at 1 kHz	55 at 1 kHz	20 at 2 kHz	50 at 1 kHz	60 at 1 kHz	52.6 at 1 kHz	55 at 1 kHz

## Data Availability

The data that support the findings of this study are available from the corresponding author upon reasonable request.

## Abbreviations

The following abbreviations are used in this manuscript:

END	Equivalent Noise Density
MEMSs	Micro-Electro-Mechanical Systems
CMOS	Complementary Metal Oxide Semiconductor
ScAlN	Scandium Aluminum Nitride
PZT	Lead Zirconate Titanate
